# Milk and Tuberculosis Disease

**Published:** 1907-03

**Authors:** 


### MILK AND TUBERCULOSIS DISEASE.

---

For a number of months now the tendency of medical opinion has been toward a decided accentuation of the dissent which was originally expressed, and more widely entertained in silence, when Robert Koch propounded the thesis that human and bovine tubercular disease differed from each other to such an extent that the propagation of the one from the other was at least uncommon. Koch's opinion on this point has met with no great subport from the first, and it is now, we feel warranted in saying, almost discredited. Much of this growth of adverse judgment has been brought about by experimental researches conducted by officers of the Bureau of Animal Industry of the United States Department of Agriculture. So far as the general public is concerned, however, it is undeniable that the widespread currency given by the newspapers to the gist of a British commission's findings within the last few days has made a tremendous impression. The commission's conclusions are quite in accord with those of our Bureau of Animal Industry, concerning which we have kept our readers informed. The essential feature of the resulting doctrine is the presumption that infection takes place much oftener by way of the digestive canal than by way of the air passages.

Now that these teachings are meeting with general acceptance, it is almost wholly to milk that the public turns its attention as the source of infection. This is both natural and justifiable, if with milk we include butter and cheese, for the other bovine food products are reasonably sure to be sterilized in the ordinary operations of the kitchen, but it will not do to lose sight altogether of the use of raw meat and its expressed juice as articles of diet. Pasteurization of milk is naturally looked to as our main reliance in avoiding infection. Probably it would answer the purpose if it were universally resorted to, but it is doubtful if, even with the present general interest in the subject, it is likely to be employed by the public on a scale large enough to check the spread of tuberculous disease materially—that is, if it is left to the people themselves. It might be practicable to require the producers or the dealers to do the pasteurizing. Apparently, it is not sufficient to rely on bacteriological examinations of milk brought to great cities, for they cannot be carried out without the consumption of enough time to allow the milk to spoil. We are inclined to think that the tuberculin test should be applied to milch cows more generally, as well as more frequently in the case of the individual cow, than has hitherto been commonly the case. Certain it is that in one way or another our milk supplies must be placed above suspicion.—Editorial, New York Medical Journal, February 9th, 1907.

